# Responsiveness of the EuroQoL 5-Dimension (EQ-5D) questionnaire in patients with spondyloarthritis

**DOI:** 10.1186/s12891-021-04315-4

**Published:** 2021-05-14

**Authors:** Helen Hoi Lun Tsang, Carlos King Ho Wong, Prudence Wing Hang Cheung, Chak Sing Lau, Ho Yin Chung, Jason Pui Yin Cheung

**Affiliations:** 1 Department of Medicine, The University of Hong Kong, Hong Kong SAR, China; 2 Department of Family Medicine and Primary Care, The University of Hong Kong, Hong Kong SAR, China; 3 Department of Orthopaedics and Traumatology, The University of Hong Kong, Professorial Block, 5th Floor, 102 Pokfulam Road, Pokfulam, Hong Kong SAR, China

**Keywords:** Spondyloarthritis, EQ-5D, SpA, HRQoL

## Abstract

**Background:**

Spondyloarthritis (SpA) has a significant impact on patients’ quality of life due to functional impairments. Generic health instruments like the EuroQoL 5-dimension (EQ-5D) is important for cost-utility analysis of health care interventions and calculation of quality-adjusted life-years. It has been validated in patients with SpA. However, its responsiveness property is unclear. Hence, the aim of study is to test the responsiveness properties of the EQ-5D health measure for Chinese patients with SpA.

**Methods:**

Prospective and consecutive recruitment of 151 Chinese patients with SpA was conducted with follow-up assessments 6 months later. Demographic data including smoking and drinking habits, education level, income and occupation was collected. Disease-associated data including disease duration, presence of back pain, peripheral arthritis, dactylitis, enthesitis, uveitis, psoriasis, and inflammatory bowel disease was also recorded. Questionnaires regarding disease activity and functional disability (BASDAI, BASFI, BASGI, BASMI, ASDAS), mental health (HADS) and the EQ-5D scores were recorded. Responsiveness was tested against the global rating of change scale (GRC) and changes in disease activity using BASDAI and ASDAS-CRP.

**Results:**

A total of 113 (74.8%) patients completed the follow-up assessments. Most patients (61.6%) had low disease activity level with BASDAI <4 and 39.7% of patients had inactive disease by ASDAS-CRP. EQ-5D scores was well discriminated along with BASDAI and BASFI scores. EQ-5D scores also correlated well with HADS. The GRC was not able to discriminate adequately. No significant ceiling or floor effect was observed.

**Conclusions:**

EQ-5D demonstrates satisfactory responsiveness property for assessment of changes in SpA disease activity.

**Level of evidence:**

II

## Background

Assessing changes in clinical status for patients with chronic illness is important. This provides clinicians and caretakers a method to gauge treatment response and deterioration in condition. The patients’ health status may not always be apparent to clinicians. Self-reported health-related quality of life (HRQoL) scores provide a subjective assessment of patient health. One chronic illness that is particularly relevant is spondyloarthritis (SpA). SpA encompasses a group of interrelated rheumatic conditions including ankylosing spondylitis (AS), psoriatic arthritis (PsA), spondyloarthritis associated with inflammatory bowel disease (IBD) and reactive arthritis. AS, regarded as the prototype of SpA, has been shown to be associated with greater work disability (WD) compared to the general population, with WD rates varying from 3-50% in western countries [1–3]. Patients with AS are 3.1 times more likely to have withdrawal from work than expected in the general population and they are also more likely to experience a lower quality of life (QoL) [4, 5]. This in turn will result in loss of work productivity and increased socioeconomic burden. Studies have also shown that patients with axial SpA report a lower HRQoL than do healthy controls and this reduction in HRQoL is associated with fatigue, pain, increased disease activity, and decreased daily activity and exercise [6–8]. In addition, a lower HRQoL in SpA patients is associated with adverse psychological outcomes and a higher prevalence of anxiety and depression [9].

There are mainly two different types of HRQoL instruments, namely disease-specific and generic, to assess patients of chronic diseases. For axial SpA, disease-specific tools for assessing functional disability include Bath Ankylosing Spondylitis Functional Index (BASFI), the Leeds Disability Questionnaire (LDQ) and the Dougados Functional Index (DFI). Generic instruments are more useful for assessments of the disease impact by allowing comparisons between different disease populations.

The EuroQoL 5-dimension (EQ-5D) is a generic health measure instrument developed by the EuroQoL group, which allows a quantitative expression of the individual’s perception of their overall health status [10]. It serves as an important utility measure for clinical and economic appraisal, particularly in the cost-utility analysis of various health care interventions, and the calculation of quality-adjusted life-years (QALYs). It has been applied to the Chinese population previously [11] and has been shown to be useful in assessing QoL in patients with SpA [12]. However, the responsiveness of EQ-5D to changes in disease status over time in patients with SpA is unclear. Responsiveness refers to the ability of a score to capture underlying changes in a patients’ health status over time. It is essential for clinicians to assess whether the treatment provided has improved the QoL in patients and whether further escalation of treatment is required. EQ-5D is also a valuable tool as it allows cross comparison with other rheumatological diseases. Hence, the aim of this study is to test the responsiveness of the EQ-5D in patients with SpA.

## Methods

A total of 151 consecutive patients of Chinese ethnicity were prospectively recruited from two rheumatology specialist clinics between May to December 2017 and subsequently reassessed at a follow-up of 6 months later (November 2017 to June 2018). All recruited patients were diagnosed to have either axial SpA or peripheral SpA by rheumatologists based on the Assessment of Spondyloarthritis international Society (ASAS) criteria [13–15] and by expert opinion. All recruited patients were 18 years old or above. Patients who did not give consent for participation, non-Chinese, illiterate and unable to comprehend the instruments were excluded. Subjects who consented were interviewed for a panel of sociodemographic and disease-associated parameters, disease activity and severity factors, and HRQoL scores that highlight the functional and mental health status. Both baseline and follow-up interviews were conducted in person at the consultation clinic. At the follow-up interview, subjects were assessed by the same research personnel for a reassessment of the same study questionnaires as well as the global rating of change scale. To provide good quality of psychometric evidence, sample size of at least 100 was recommended by Terwee *et al* [16]. Ethics was approved by the local institutional review board. All methods were carried out in accordance with relevant guidelines and regulations.

### Sociodemographic and disease-associated data

Patients’ smoking and drinking habits, education level, income and occupation were recorded. Disease-associated data including disease duration, presence of back pain and/or peripheral arthritis, dactylitis, enthesitis, and extra-articular manifestations such as uveitis, psoriasis, and IBD were collected. Baseline treatment including the use of non-steroidal anti-inflammatory drugs (NSAIDs) or cyclooxygenase-2 (cox-2) inhibitors, disease modifying anti-rheumatic drugs (DMARDs) and biologics and any subsequent change in treatment after 6 months were documented. Physical examination was performed to determine the number of tender joint count and swollen joint count, the dactylitis and enthesitis scores. Antero-posterior radiograph of the lumbosacral spine was utilized for grading of sacroiliitis according to the modified New York criteria [17] by a rheumatologist (HYC) who was blinded to the clinical data. Radiological sacroiliitis was graded as: 0, normal; 1, suspicious; 2, minimal sclerosis with some erosions; 3, erosion with widening of joint space and possible partial ankyloses; 4, complete ankyloses. Bilateral sacroiliitis of grade 2 or above, or unilateral sacroiliitis of grade 3 or above was defined as AS. Patients were treated by the attending rheumatologist according to their disease activity and severity.

### Disease activity and severity scores

All recruited patients filled in the Bath Ankylosing Spondylitis Disease Activity Index (BASDAI) [18] and BASFI [19] to determine the disease activity and functional disability respectively. Spinal mobility was assessed clinically to determine the Bath Ankylosing Spondylitis Metrology Index (BASMI) score [20]. The Bath Ankylosing Spondylitis Global Index (BASGI) [21] and C-reactive protein (CRP) were measured for calculation of the Ankylosing Spondylitis Disease Activity Score-CRP (ASDAS-CRP) [22], which is a composite disease activity measure of SpA. Human leucocyte antigen (HLA) B27 status was also checked as a poor prognostic marker. BASDAI and ASDAS are more often used for patients with axial disease. However, both tools have demonstrated good discriminatory ability in patients with peripheral SpA as well [23].

### Functional and mental health status

The SF-36 [24–26] was used for assessment of mental and physical health and as a comparable generic questionnaire marker of EQ-5D changes. Hospital Anxiety and Depression Scale (HADS) [27] is a fourteen-item scale with seven items each for anxiety and depression subscales. It has been validated in Chinese axial SpA patients and is found to useful in screening for depressive and anxiety disorders in SpA [28].

The main study parameter was the EQ-5D which is a standardized measure of health status developed by the EuroQoL group that allows a generic assessment of health status for clinical and economic appraisal [10]. It has been useful in assessing the HRQoL in patients with musculoskeletal problems [29–33]. It consists of a 2-page questionnaire, the EQ-5D descriptive system and the EQ visual analogue scale (EQ VAS). The descriptive system is comprised of 5 domains, including mobility, self-care, usual activities, pain/discomfort and anxiety/depression. There are 2 versions of EQ-5D, namely the EQ-5D-3 level (EQ-5D-3L) and the EQ-5D-5 level (EQ-5D-5L) versions. For the EQ-5D-3L, each domain will be scored by 3 levels (no problem, some problem and extreme problem). We utilized the EQ-5D-5L version for this study and each domain of this parameter was scored by 5 levels with 1 representing no problem and 5 representing extreme problem. Previous studies published by EuroQoL group have shown that the 5 level version could significantly increase reliability and sensitivity while maintaining the feasibility of the test and it could potentially reduce ceiling effects [10]. The scores of the 5 domains are combined into a 5-digit number which is converted into a single index value. The EQ-VAS allows patients to self-report their own perceived quality of life from a scale of 0 (worst) to 100 (best). We applied Chinese-specific EQ-5D-5L value set ranging from -0.391 for the worst health status (‘55555’) to 1 for the best health status (‘11111’) to estimate EQ score [34].

### Generic and Clinical Anchors

It was necessary to include an external anchor to act as a reference for indicating patient improvement or deterioration. To test the responsiveness of EQ-5D, this anchor represented the patient-reported assessment of health change over time and thus indicate whom change in health occurred [35]. The global rating of change (GRC) scale is a single-item outcome measure for independent scoring of self-perceived improvement in a patient retrospectively and has been used in musculoskeletal research [36]. All subjects answered the question “Compared to the previous visit, how would you rate your overall health now?” [36]. The response scale was a seven-point Likert scale ranging from -3 to 3 corresponding to the ‘much worse’ to the ‘much better’ options with 0 for ‘no change’. Three groups were defined using this scale: ‘worse’ (-3 to -1), ‘unchanged’ (0) and ‘improved’ (1 to 3) and such re-grouping or categorization was applied in previous studies to evaluate responsiveness [30, 37].

The GRC was a generic anchor used to test the overall patient improvement or deterioration. Clinical anchors were also applied namely BASDAI and ASDAS-CRP to assess the changes in disease activity. These differences were more representative of actual improvement or deterioration in the disease as compared to the GRC scale which may be subjected to mental and psychological influences.

### Statistical analysis

Overall descriptive characteristics were reported with mean ± standard deviation (SD). Any differences between baseline and follow-up were compared using independent t-test and Chi-squared test where appropriate. The responsiveness of the EQ-5D was assessed using the effect size statistics. Differences between baseline and follow-up of the utility score was evaluated by standardized effect size (SES) and standardized response mean (SRM) separately for GRC, BASDAI, BASFI and ASDAS-CRP. We have adopted the minimum clinically important improvement (MCII) of 1.1 for BASDAI and 0.6 for BASFI [38]. Change in the MCII of BASDAI and BASFI will be correlated with change in EQ-5D. As for ASDAS-CRP, it is categorized as inactive disease (<1.3), moderate disease activity (1.3-<2.1), high disease activity (2.1-<3.5), and very high disease activity (>3.5). A change of 1.1 is considered as clinically significant change [39]. The SES and SRM results were interpreted as trivial for values <0.2, small for values ≥0.2 to <0.5, moderate for values ≥0.5 to <0.8, and large for values ≥0.8 [40]. Differences in mean change at follow-up by disease activity assessment with BASDAI and BASFI, and GRC, were performed along with area under the curve analysis (AUC).

Spearman’s correlation was performed to assess the relationship between changes in EQ-5D scores with erythrocyte sedimentation rate (ESR), C-reactive protein (CRP), ASDAS-CRP, ASDAS-ESR, BASDAI, BASFI, SF-36, and HADS. Spearman’s correlation was used because the data was not normally distributed as reviewed by the Shapiro-Wilk normality test. The correlation coefficient is considered weak at 0.3, moderate at 0.5 and strong at 0.7. All statistical analyses were conducted using STATA version 13.0. A p-value of <0.05 was considered as statistically significant and 95% confidence intervals (CIs) were listed as appropriate.

## Results

From a total of 151 Chinese patients with SpA recruited consecutively at baseline, 113 (74.8%) completed the follow-up assessments. The baseline demographics are listed in Table 1. The mean age of subjects who completed all assessments was 44.7±13.0 years, and 66.4% of them were male patients. Most patients (61.6%) had low disease activity with BASDAI of <4 and 39.7% of patients had inactive disease by ASDAS-CRP. For the baseline treatment, 75.5% of the patients were on NSAIDs or cox-2 inhibitors, 31.8% were on DMARDs (including sulphasalazine, methotrexate and/or leflunomide) and 25.8% were on biologics (including tumour necrosis factor inhibitors, secukinumab or ustekinumab).

**Table 1:** Demographic and clinical characteristics of patients

		Baseline (*N*=151)	Follow-up Completion (*N*=113)	Follow-up Incompletion (*N*=38)	*P*-value
**Demographic, % (n)**					
Age, mean ± SD		45.5±13.0	44.7±13.0	47.6±12.7	0.234
Gender					0.145
	Female	30.5 % (46)	33.6 % (38)	21.1 % (8)	
	Male	69.5 % (105)	66.4 % (75)	78.9 % (30)	
Smoking					0.011*
	Non-smoker	81.5 % (123)	86.7 % (98)	65.8 % (25)	
	Smoker	9.9 % (15)	6.2 % (7)	21.1 % (8)	
	Ex-smoker	8.6 % (13)	7.1 % (8)	13.2 % (5)	
Drinking					0.732
	Non-drinker	29.3 % (44)	30.4 % (34)	26.3 % (10)	
	Ex-drinker	11.3 % (17)	9.8 % (11)	15.8 % (6)	
	Social drinker	55.3 % (83)	55.4 % (62)	55.3 % (21)	
	Current drinker	4.0 % (6)	4.5 % (5)	2.6 % (1)	
Education level					0.009*
	Primary	8.0 % (12)	9.8 % (11)	2.6 % (1)	
	Secondary	47.3 % (71)	40.2 % (45)	68.4 % (26)	
	Tertiary or above	44.7 % (67)	50.0 % (56)	29.0 % (11)	
Family income level					0.550
	<HK$10000	18.0 % (27)	17.9 % (20)	18.4 % (7)	
	HK$10000-30000	43.3 % (65)	41.1 % (46)	50.0 % (19)	
	HK$30000-60000	20.7 % (31)	20.5 % (23)	21.1 % (8)	
	>HK$60000	18.0 % (27)	20.5 % (23)	10.5 % (4)	
Occupation					0.905
	Student	7.3 % (11)	8.0 % (9)	5.3 % (2)	
	Housewife	5.3 % (8)	4.4 % (5)	7.9 % (3)	
	Work	72.9 % (110)	72.6 % (82)	73.7 % (28)	
	Unemployed	3.3 % (5)	3.5 % (4)	2.6 % (1)	
	Retired	11.3 % (17)	11.5 % (13)	10.5 % (4)	
**Clinical, % (n)**					
Positive HLA-B27					0.267
	No	18.7 % (26)	20.8 % (22)	12.1 % (4)	
	Yes	81.3 % (113)	79.2 % (84)	87.9 % (29)	
BASDAI					0.588
	Low disease activity (<4)	61.6 % (93)	62.8 % (71)	57.9 % (22)	
	High disease activity (>=4)	38.4 % (58)	37.2 % (42)	42.1 % (16)	
ASDAS-CRP					<0.001*
	Inactive disease (<1.3)	39.7 % (60)	47.8 % (54)	15.8 % (6)	
	Moderate disease activity (1.3-2.1)	30.5 % (46)	30.1 % (34)	31.6 % (12)	
	High disease activity (2.1-3.5)	26.5 % (40)	21.2 % (24)	42.1 % (16)	
	Very high disease activity (>3.5)	3.3 % (5)	0.9 % (1)	10.5 % (4)	
Family History					0.655
	No	71.8 % (107)	71.2 % (79)	73.7 % (28)	
	Yes	26.8 % (40)	27.9 % (31)	23.7 % (9)	
Axial spondyloarthritis					0.078
	No	17.2 % (26)	20.4 % (23)	7.9 % (3)	
	Yes	82.8 % (125)	79.6 % (90)	92.1 % (35)	
Peripheral spondyloarthritis					0.030*
	No	83.4 % (126)	79.6 % (90)	94.7 % (36)	
	Yes	16.6 % (25)	20.4 % (23)	5.3 % (2)	
Peripheral arthritis					0.093
	No	62.3 % (94)	58.4 % (66)	73.7 % (28)	
	Yes	37.7 % (57)	41.6 % (47)	26.3 % (10)	
Dactylitis					0.787
	No	96.7 % (146)	96.5 % (109)	97.4 % (37)	
	Yes	3.3 % (5)	3.5 % (4)	2.6 % (1)	
Uveitis					0.872
	No	64.2 % (97)	64.6 % (73)	63.2 % (24)	
	Yes	35.8 % (54)	35.4 % (40)	36.8 % (14)	
Psoriasis					
	No	87.4 % (132)	85.0 % (96)	94.7 % (36)	
	Yes	12.6 % (19)	15.0 % (17)	5.3 % (2)	
Inflammatory Bowel Disease					0.994
	No	97.4 % (147)	97.3 % (110)	97.4 % (37)	
	Yes	2.6 % (4)	2.7 % (3)	2.6 % (1)	
HADS					
	Depression				0.124
	Normal (0-7)	80.6 % (112)	79.8 % (83)	82.9 % (29)	
	Borderline (8–10)	14.4 % (20)	14.4 % (15)	14.3 % (5)	
	Abnormal (11–21)	5.0 % (7)	5.8 % (6)	2.9 % (1)	
	Anxiety				0.407
	Normal (0-7)	68.4 % (95)	71.2 % (74)	60.0 % (21)	
	Borderline (8–10)	20.9 % (29)	19.2 % (20)	25.7 % (9)	
	Abnormal (11–21)	10.8 % (15)	9.6 % (10)	14.3 % (5)	
Backpain duration		16.6±12.1	15.9±11.5	17.4±12.7	0.540
Current back pain					0.285
	No	22.0 % (33)	24.1 % (27)	15.8 % (6)	
	Yes	78.0 % (117)	75.9 % (85)	84.2 % (32)	
Tender joints		0.36±1.23	0.40±1.36	0.24±0.75	0.488
Swollen joints		0.21±1.12	0.25±1.28	0.08±0.36	0.424
Dactylitis score		0.01±0.11	0.01±0.09	0.03±0.16	0.419
Enthesitis score		0.23±0.77	0.28±0.86	0.08±0.36	0.158
**Baseline treatment**					
NSAIDs/COX-2 Inhibitors					0.892
	No	24.5 % (37)	24.8 % (28)	23.7 % (9)	
	Yes	75.5 % (114)	75.2 % (85)	76.3 % (29)	
DMARDs					0.041*
	No	68.2 % (103)	63.7 % (72)	81.6 % (31)	
	Yes	31.8 % (48)	36.3 % (41)	18.4 % (7)	
Biologics					0.102
	No	74.2 % (112)	70.8 % (80)	84.2 % (32)	
	Yes	25.8 % (39)	29.2 % (33)	15.8 % (6)	
**Baseline disease activity status**					
	BASDAI	3.45±1.91	3.43±1.90	3.52±1.95	0.793
	BASFI	2.13±1.98	2.09±2.06	2.28±1.75	0.610
	BASMI	4.05±1.63	3.98±1.56	4.29±1.82	0.309
	ASDAS-ESR	2.36±0.92	2.34±0.93	2.42±0.88	0.655
	ASDAS-CRP	1.63±0.98	1.42±0.89	2.26±0.98	<0.001
	CRP (g/dL)	0.76±1.57	0.75±1.63	0.81±1.36	0.846
	ESR (mm/hr)	23.26±18.21	23.37±19.47	22.95±14.04	0.902
**SF-36**					
	Physical functioning	75.97±19.90	75.88±19.70	76.25±20.75	0.923
	Role limitations due to physical functioning	70.62±25.36	69.38±25.53	74.18±24.84	0.317
	Bodily pain	33.64±8.68	33.34±9.01	34.54±7.65	0.468
	General health perceptions	54.76±9.27	54.09±9.40	56.73±8.72	0.135
	Vitality	53.93±11.51	53.75±10.55	54.44±14.08	0.751
	Role limitations due to emotional problems	72.35±23.73	72.50±23.77	71.93±23.92	0.899
	Social functioning	48.92±8.92	47.99±8.48	51.64±9.71	0.029*
	Mental health	56.17±10.62	56.21±10.76	56.05±10.34	0.938
	Physical component summary	38.24±7.24	37.97±7.27	39.03±7.20	0.696
	Mental component summary	42.76±5.34	42.75±5.24	42.77±5.71	0.988
**HADS**					
	Anxiety	5.91±3.60	5.76±3.61	6.37±3.58	0.386
	Depression	4.81±3.46	4.71±3.56	5.11±3.17	0.553
	Total	10.70±6.59	10.43±6.68	11.49±6.32	0.415

*HLA-B27* Human leukocyte antigen B27; *BASDAI* Bath Ankylosing Spondylitis Disease Activity Index; *BASFI* Bath Ankylosing Spondylitis Functional Index; *BASMI* Bath Ankylosing Spondylitis Metrology Index; *ASDAS* Ankylosing Spondylitis Disease Activity Score; *CRP* C-reactive protein; *ESR* Erythrocyte sedimentation rate; *HADS* Hospital Anxiety and Depression Scale; *NSAIDs* Non-steroidal anti-inflammatory drugs; *COX-2* Cyclooxygenase-2; *DMARDs* Disease-modifying antirheumatic drugs; *SF-36* 36-item short form questionnaire

*statistically significant (*p*<0.05)

**Fig. 1:**
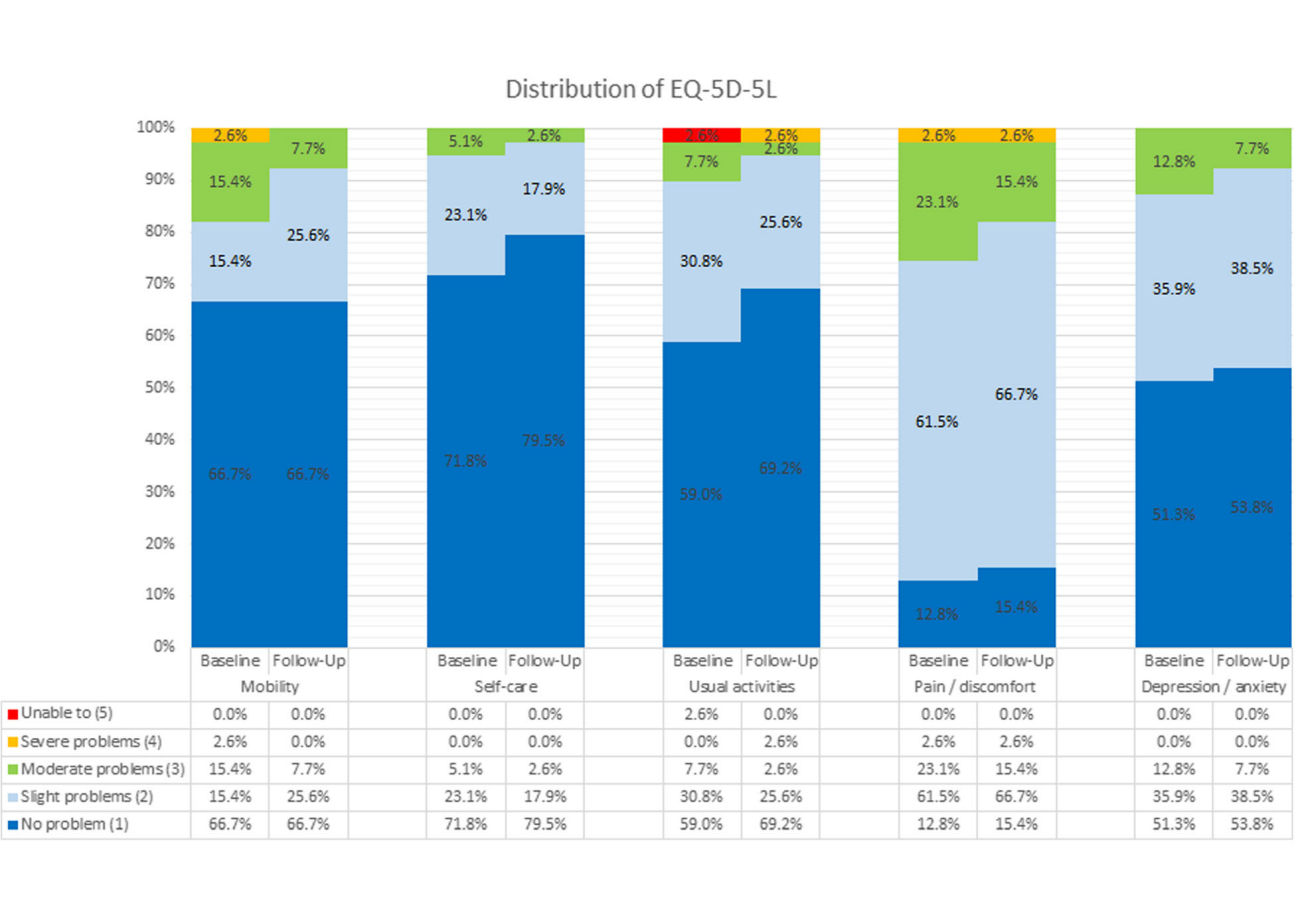
Distribution of EQ-5D-5L responses in the study cohort

The mean change of EQ-5D and EQ-VAS scores by disease activity and GRC are shown in Tables 2 and 3. Improved and worsened EQ-5D scores discriminated well with change in disease activity level measured by BASDAI (improved: *p*=0.012, SES=0.84, SRM=0.87, RS=1.05; worsened: *p*=0.004, SES=-0.70, SRM=1.00, RS=-0.74). Using the MCII, the EQ-5D scores discriminated well with BASDAI (*p*=0.001, SES=1.07, SRM=1.19, RS=1.03) and with BASFI (*p*=0.001, SES=0.79, SRM=1.12, RS=0.73). Post-hoc power analysis showed that sample sizes of 13 in a group of worsened disease activity measured by BASDAI achieved 96% to detect a difference of -0.08 with an estimated SD of 0.08 and a significance 0.05 using one-sided one sample t-test, and sample size of 12 in an improved group achieved 86% to detect a difference of 0.11 assuming an estimated SD of 0.13 using one-sided one sample t-test. Up to 88 patients did not have a change in disease activity level based on BASDAI. For BASDAI and BASFI MCII, the mean difference detected was 0.16 and 0.15 respectively. The effect size (1.36 and 1.29) and AUC (0.85 and 0.83) were acceptable. There were no patients listed as clinically improved with the ASDAS-CRP. No significant findings were observed for the GRC. When comparing the EQ-5D scores at baseline and follow-up, no significant ceiling or floor effects were observed (Table 4). Comparing the differences in EQ-5D-5L scores from baseline to follow-up (Fig. 1), there was overall improvement in various domains: mobility (31.4% with one level reduction), usual activities (22.9% with one level reduction), pain/discomfort (22.9% with one level reduction), depression/anxiety (17.1% with one level reduction) and self-care (17.1% with one level reduction). Change in EQ-5D score correlates with changes in the SF36 domains of physical function (*r*=-0.202; *p*=0.036), role limitation due to physical function (*r*=-0.205; *p*=0.033) and role limitation due to emotional problems (*r*=-0.247; *p*=0.009). Similarly, change in EQ-5D scores significantly correlated with both anxiety and depression domains of HADS. There was no correlation between the change in EQ5D scores and change in treatment at 6 months but the addition of NSAIDs/cox-2 inhibitor was significantly associated with improvement in EQ-VAS score (Table 5).

**Table 2:** Mean Change, Standardized Effect Size, Standardized Response Mean and Responsiveness Statistic of EQ-5D Score and EQ-VAS by Disease Activity and GRS

Measure/subscale	Baseline (Mean±SD)	At follow-up (Mean±SD)	Paired difference (Mean±SD)	*P*-value	SES (95% CI)	SRM (95% CI)	RS (95% CI)
**Disease activity measured by BASDAI**							
Worsened group (*N*=13)							
EQ-5D score	0.79±0.11	0.71±0.14	-0.08±0.08	0.004*	-0.70 (-1.05,-0.31)	-1.00 (-1.51,-0.45)	-0.74 (-1.12,-0.33)
EQ-VAS	56.92±17.02	54.46±13.56	-2.46±15.45	0.576	-0.14 (-0.70,0.27)	-0.16 (-0.77,0.30)	-0.12 (-0.56,0.22)
Unchanged group (*N*=88)							
EQ-5D score	0.81±0.18	0.80±0.20	-0.01±0.11	0.435	-0.05 (-0.17,0.08)	-0.08 (-0.29,0.13)	-0.08 (-0.29,0.13)
EQ-VAS	66.69±17.39	65.31±19.01	-1.39±21.34	0.544	-0.08 (-0.36,0.16)	-0.06 (-0.29,0.13)	-0.06 (-0.29,0.13)
Improved group (*N*=12)							
EQ-5D score	0.71±0.13	0.83±0.11	0.11±0.13	0.012*	0.84 (0.42,1.48)	0.87 (0.44,1.54)	1.05 (0.53,1.86)
EQ-VAS	60.25±13.46	67.17±11.61	6.92±13.61	0.106	0.51 (-0.09,1.00)	0.51 (-0.09,0.99)	0.32 (-0.05,0.63)
**Clinical improvement measured by MCII of BASDAI**							
Clinically improved group (*N*=15)							
EQ-5D score	0.69±0.14	0.84±0.13	0.15±0.12	0.001*	1.07 (0.47,1.81)	1.19 (0.55,1.67)	1.03 (0.48,1.60)
EQ-VAS	56.20±18.18	65.40±14.8	9.20±20.64	0.106	0.55 (-0.05,1.29)	0.45 (-0.13,1.06)	0.51 (-0.06,1.13)
Unchanged or no clinical improvement (*N*=98)							
EQ-5D score	0.81±0.17	0.80±0.18	-0.01±0.11	0.287	-0.07 (-0.21,0.06)	-0.11 (-0.34,0.08)	-0.07 (-0.21,0.06)
EQ-VAS	66.21±16.75	64.08±18.57	-2.13±19.71	0.282	-0.12 (-0.34,0.08)	-0.11 (-0.30,0.09)	-0.13 (-0.38,0.09)
**Clinical improvement measured by MCII of BASFI**							
Clinically improved group (*N*=16)							
EQ-5D score	0.62±0.19	0.76±0.17	0.14±0.13	0.001*	0.79 (0.45,1.23)	1.12 (0.74,1.48)	0.73 (0.41,1.22)
EQ-VAS	54.06±19.81	62.81±14.80	8.75±23.13	0.151	0.50 (-0.31,1.12)	0.38 (-0.24, 1.01)	0.44 (-0.27,1.02)
Unchanged or no clinical improvement (*N*=97)							
EQ-5D score	0.83±0.15	0.81±0.17	-0.01±0.11	0.268	-0.08 (-0.22,0.06)	-0.11 (-0.32,0.09)	-0.08 (-0.24,0.06)
EQ-VAS	66.67±16.16	64.49±18.61	-2.18±19.27	0.269	-0.12 (-0.36,0.10)	-0.11 (-0.31,0.09)	-0.13 (-0.42,0.10)
**Disease activity measured by ASDAS-CRP**							
Clinically improved group (*N*=0)							
EQ-5D score							
EQ-VAS							
Not clinically improved group (*N*=113)							
EQ-5D score	0.80±0.17	0.79±0.18	0.00±0.11	0.706	-0.02 (-0.15,0.10)	-0.04 (-0.22,0.16)	
EQ-VAS	64.88±17.21	64.26±18.07	-0.63±20.11	0.740	-0.04 (-0.26,0.16)	-0.03 (-0.22,0.14)	
**Global rating of change**							
Worsened group (*N*=39)							
EQ-5D score	0.78±0.13	0.77±0.14	-0.01±0.10	0.754	-0.04 (-0.26,0.21)	-0.05 (-0.34,0.28)	-0.05 (-0.31,0.26)
EQ-VAS	62.90±15.53	58.49±14.83	-4.41±20.33	0.184	-0.28 (-0.72,0.10)	-0.22 (-0.55,0.08)	-0.21 (-0.53,0.07)
Unchanged group (*N*=35)							
EQ-5D score	0.80±0.18	0.79±0.19	-0.01±0.11	0.671	-0.04 (-0.24,0.15)	-0.07 (-0.40,0.25)	-0.07 (-0.40,0.25)
EQ-VAS	66.69±18.65	60.00±19.89	-6.69±21.04	0.069	-0.36 (-0.76,0.00)	-0.32 (-0.67,0.00)	-0.32 (-0.67,0.00)
Improved group (*N*=24)							
EQ-5D score	0.83±0.17	0.86±0.12	0.03±0.13	0.286	0.17 (-0.08,0.55)	0.22 (-0.10,0.73)	0.26 (-0.12,0.85)
EQ-VAS	68.96±15.32	77.33±13.15	8.38±14.80	0.011*	0.55 (0.22,1.00)	0.57 (0.23,1.03)	0.40 (0.16,0.72)

*BASDAI* Bath Ankylosing Spondylitis Disease Activity Index; *ASDAS* Ankylosing Spondylitis Disease Activity Score; *CRP* C-reactive protein; *EQ-5D* EuroQol 5-Dimension; *EQ-VAS* EuroQol Visual Analogue Scale; *MCII* Minimum Clinically Important Improvement

^*^ denotes statistical significance

**Table 3:** Difference in Mean Change at follow-up by Disease activity (BASDAI), Clinical Improvement (Minimum Clinically Important Improvement (MCII)) and Global Rating of Change Scale

**Disease activity**	**Change in EQ-5D score**			**Change in EQ-VAS**		
	Mean Difference (95% CI)	Effect size	AUC (95% CI)	Mean Difference (95% CI)	Effect size	AUC (95% CI)
Unchanged Vs Worsened	0.07 (0.01,0.13)	0.66	0.74 (0.59,0.88)	1.08 (-11.14,13.29)	0.05	0.52 (0.36,0.68)
Improved Vs Unchanged	0.12 (0.05,0.19)	1.05	0.80 (0.67,0.94)	8.30 (-4.29,20.89)	0.40	0.63 (0.46,0.79)
Improved Vs Worsened	0.19 (0.10,0.28)	1.35	0.93 (0.82,1.00)	9.38 (-2.71,21.47)	0.62	0.68 (0.46,0.90)
Improved/unchanged Vs worsened	0.08 (0.02,0.15)	0.74	0.76 (0.62,0.89)	2.07 (-9.73,13.87)	0.10	0.54 (0.38,0.69)
Improved Vs worsen/unchanged	0.13 (0.06,0.19)	1.13	0.82 (0.69,0.95)	8.44 (-3.68,20.56)	0.42	0.64 (0.47,0.80)
	**Change in EQ-5D score**			**Change in EQ-VAS**		
Clinical improvement	Mean Difference (95% CI)	Effect size	AUC (95% CI)	Mean Difference (95% CI)	Effect size	AUC (95% CI)
Based on BASDAI MCII						
Improved Vs Not Improved	0.16 (0.10,0.22)	1.36	0.85 (0.75,0.95)	11.33 (0.44,22.23)	0.56	0.65 (0.49,0.82)
Based on BASFI MCII						
Improved Vs Not Improved	0.15 (0.09,0.21)	1.29	0.83 (0.74,0.92)	10.9 (0.32,21.53)	0.51	0.67 (0.52,0.83)
**Global Rating of Change Scale**	**Change in EQ-5D score**			**Change in EQ-VAS**		
	Mean Difference (95% CI)	Effect size	AUC (95% CI)	Mean Difference (95% CI)	Effect size	AUC (95% CI)
Unchanged Vs Worsened	-0.00 (-0.05,0.05)	0.03	0.52 (0.40,0.65)	-2.28 (-11.87,7.32)	0.11	0.48 (0.35,0.60)
Improved Vs Unchanged	0.04 (-0.03,0.10)	0.31	0.52 (0.38,0.66)	15.06 (5.10,25.02)	0.75	0.69 (0.56,0.82)
Improved Vs Worsened	0.03 (-0.02,0.09)	0.30	0.55 (0.42,0.69)	12.79 (3.22,22.35)	0.66	0.66 (0.53,0.78)
Improved/unchanged Vs worsened	0.01 (-0.03,0.06)	0.11	0.53 (0.42,0.65)	3.85 (-4.41,12.11)	0.19	0.55 (0.44,0.67)
Improved Vs worsen/unchanged	0.04 (-0.02,0.09)	0.32	0.54 (0.42,0.66)	13.86 (4.85,22.88)	0.69	0.67 (0.56,0.78)

*EQ-5D* EuroQol 5-dimension; *EQ-VAS* EuroQol Visual Analogue Scale; *CI* confidence interval; *AUC* Area under the curve; *MCII* Minimum Clinically Important Improvement

**Table 4:** Descriptive statistics of EQ-5D-5L utility score and EQ-VAS at baseline and follow-up

	Mean	Standard deviation	Observed range	Theoretical range	Floor (%)	Ceiling (%)
EQ-5D-5L						
Baseline	0.798	0.170	0.280 to 1.000	− 0.391 to 1.000	0.0	6.2
Baseline^a^	0.798	0.162	0.310 to 1.000	− 0.391 to 1.000	0.0	5.1
Follow-up	0.794	0.185	-0.110 to 1.000	− 0.391 to 1.000	0.0	13.3
Mean change	-0.004	0.114	-0.390 to 0.410			
EQ-VAS						
Baseline	64.88	17.21	10 to 97	0 to 100	0.0	0.0
Baseline^a^	65.73	16.68	15 to 97	0 to 100	0.0	0.0
Follow-up	64.26	18.07	5 to 95	0 to 100	0.0	0.0
Mean change	-0.63	20.11	-60 to 50			

^a^Baseline descriptive statistics of respondents who have completed both baseline and follow-up

*EQ-5D-5L* EuroQol 5-dimension 5-level; *EQ-VAS* EuroQol visual analogue scale

**Table 5:** Change of treatment status at time of follow-up in relation to EQ-5D

At follow-up		% (n)	EQ-5D scores		Change of EQ-5D scores	*p*-value^a^	EQ-VAS		Change of EQ-VAS	*p*-value^a^
			Baseline	Follow-up			Baseline	Follow-up		
Change of treatment	Yes	13.2% (20)	0.76 (0.21)	0.75 (0.26)	-0.03 (0.13)	0.742	64.75 (19.27)	65.25 (21.18)	0.50 (17.06)	0.870
	No	86.8% (131)	0.80 (0.18)	0.80 (0.17)	0.00 (0.11)		63.74 (17.85)	65.12 (17.70)	1.42 (21.10)	
Change of biologics	Yes	2.6% (4)	0.71 (0.29)	0.81 (0.06)	-0.05 (0.11)	0.459	58.00 (32.29)	63.75 (24.96)	5.75 (14.57)	0.644
	No	97.4% (147)	0.80 (0.18)	0.79 (0.19)	0.00 (0.11)		64.03 (17.60)	65.18 (18.01)	1.18 (20.72)	
Change of NSAIDs/COX-2 Inhibitors	Yes	1.3% (2)	0.71 (0.24)	0.75 (0.28)	0.04 (0.04)	0.236	52.50 (24.75)	55.00 (21.21)	2.50 (3.54)	0.889
	No	98.7% (149)	0.80 (0.18)	0.79 (0.18)	0.00 (0.11)		64.03 (17.94)	65.28 (18.12)	1.28 (20.70)	
Change of DMARDs	Yes	2.0% (3)	0.81 (0.10)	0.87 (0.05)	0.10 (0.14)	0.262	66.67 (15.28)	66.67 (20.82)	0.00 (10.00)	0.835
	No	98.0% (148)	0.80 (0.18)	0.79 (0.19)	-0.01 (0.11)		63.82 (18.07)	65.11 (18.14)	1.33 (20.73)	
Addition of NSAIDs/COX-2 Inhibitors	Yes	2.0% (3)	0.72 (0.38)	0.59 (0.61)	-0.13 (0.23)	0.340	65.67 (13.65)	86.67 (7.64)	21.00 (8.54)	0.039*
	No	98.0% (148)	0.80 (0.18)	0.80 (0.16)	0.00 (0.11)		63.84 (18.09)	64.70 (18.01)	0.90 (20.55)	
Addition of DMARDs	Yes	2.0% (3)	0.90 (0.06)	0.91 (0.04)	0.03 (0.04)	0.579	78.33 (7.64)	56.67 (32.15)	-21.67 (25.66)	0.081
	No	98.0% (148)	0.79 (0.18)	0.79 (0.18)	0.00 (0.11)		63.58 (18.02)	65.31 (17.87)	1.77 (20.28)	
Addition of biologics	Yes	4.0% (6)	0.76 (0.14)	0.72 (0.11)	-0.05 (0.06)	0.123	66.00 (16.31)	65.83 (18.55)	-0.17 (10.68)	0.672
	No	96.0% (145)	0.80 (0.18)	0.80 (0.19)	0.00 (0.12)		63.78 (18.10)	65.11 (18.17)	1.36 (20.89)	

*NSAIDs* non-steroidal anti-inflammatory drugs, *COX-2* Cyclooxygenase-2; *DMARDs* Disease-modifying anti-rheumatic drugs (including sulphasalazine, methotrexate, leflunomide); *EQ-5D* EuroQol 5-dimension; *EQ-VAS* EuroQol visual analogue scale

^a^ Mann-Whitney U test for comparison of change of scores/VAS between Yes and No groups *denotes statistical significance *p*<0.05

## Discussion

SpA is a chronic debilitating disease that significantly reduces a patient’s QoL. The disease cannot be eradicated and thus patients require prolonged treatment to control the disease process and reduce symptomatology. Constant monitoring is necessary as symptoms and disease activity may fluctuate and warrant prompt adjustment of medications. This carries a heavy toll on patients’ physical and mental wellness as they are faced with changing treatment outcomes, for better or worse, and facing new concerns and complications. With the high cost for various disease-modifying drugs, it is important for the patients and medical practitioners to design the most cost-effective strategies. Determining QALYs aid in this understanding of disease burden on the healthcare system, which will in turn drive various institutional policies based on cost-utility analyses. The EQ-5D has been shown to be an effective utility score for SpA. We have found the EQ-5D to discriminate improved and worsened disease activity levels well in patients with SpA.

The EQ-5D instrument is a good measure of disease activity change as shown by its strong association with clinically significant changes in BASDAI and BASFI scores shown by the SES and SRM. The SES was near 0 in the unchanged group which verifies its accuracy in detecting change. The SES of EQ-5D for the improved group was 0.84 and for the worsened group was -0.70. These results were similar to that of other chronic musculoskeletal disorders like scoliosis deformities [37]. The higher disease activities supported by increased BASDAI score was identified by a reduction in EQ-5D. Similarly, reduced disease activity shown by reduced BASDAI score is matched by an increased EQ-5D score. No change in disease activity was also supported by no change in EQ-5D scores. Despite a small percentage of individuals with a ceiling effect at baseline and follow-up, the scores are representative of disease status changes. The ceiling effect indicates the highest possible score on the instrument and normally refers to clustering of scores at a certain extreme. This corresponded to the low disease activity scores that are unlikely to experience further improvement in health at follow-up. Conversely, there is no floor effect indicating that the instrument is sensitive to deteriorations in disease status that warrants treatment regimen changes. Hence, the EQ-5D is an appropriate tool for studying patients with SpA.

Due to the lack of clinically improved patients by ASDAS-CRP, we were unable to formulate any useful conclusions. This may be the limitation of its score to detect patient perceived QoL. Although we followed the clearly established cut-off value of ASDAS-CRP to determine improvement or worsened scores [39], we were unable to identify any individuals with improved ASDAS-CRP despite improved patients categorized by BASDAI. Despite ASDAS-CRP being a more objective assessment of SpA disease activity, it may not reflect the patient’s perceived health as well. The components of BASDAI describes more subjective self-perceived components of pain, discomfort, and other disease manifestations. Hence, it is expected for BASDAI and EQ-5D to match better since they are both patient perceived HRQoL scores.

It is also interesting to see the GRC scale as an unsatisfactory anchor for EQ-5D changes. This is not an unusual finding. Some HRQoL measures may be more responsive to a clinical anchor rather than GRC [41]. Moreover, the GRC may be affected by many other factors whereas BASDAI is more targeted to the various facets of the disease. Various external factors such as the rapport with the doctor and mental status of the patient may influence the reporting of GRC. Comparatively, BASDAI is more appropriate for the disease status and it appears that the EQ-5D is able to capture changes in disease status as well.

We also found a significant correlation between EQ-5D and HADS. HADS is a tool commonly used by psychiatrists for assessing risks of depression and anxiety. Various studies have demonstrated a prevalence of depression ranging from 11 to 31% in patients with SpA [28, 42, 43]. By correlating EQ-5D with the HADS scores, we can associate a worse HRQoL in the presence of anxiety and depression. The EQ-5D score can also help identify patients with a higher risk of developing depression and anxiety. We found that the addition of NSAIDs or cox-2 inhibitors correlate with an improvement in EQ-VAS but it did not correlate with the change in EQ-5D scores. This suggests that pain reduction is not the sole determinant of HRQoL for patients with SpA.

The main limitation of this study is an incomplete follow-up of 25%. Nevertheless, the proportion of disease activity categories and patient profiles remain similar. There is also sample heterogeneity with variable presentations of axial or peripheral involvement. Nevertheless, we did have a reasonable effect size generated from the EQ-5D results. It is also important to note that the disease-specific Assessment of SpondyloArthritis international Society (ASAS) health index [44] was not used in this study. This is an instrument that should be compared with EQ-5D in future study.

## Conclusion

The EQ-5D-5L demonstrates satisfactory responsiveness properties for assessment of changes in health status in patients with SpA. It appears to represent the patient reported HRQoL better than more objective assessments. Future study should assess the versatility of the utility score to compare different treatment regimens and its cost-utility with other chronic diseases.

## Data Availability

The datasets used and/or analyzed during the current study are available from the corresponding author on reasonable request. They are not publicly available as they are currently part of a prospective cohort that will be used for future analyses.

## Abbreviations

AS: Ankylosing spondylitis
ASDAS: Ankylosing spondylitis disease activity score
AUC: Area under the curve
BASDAI: Bath ankylosing spondylitis disease activity Index
BASFI: Bath ankylosing spondylitis functional index
BASGI: Bath ankylosing spondylitis global index
BASMI: Bath ankylosing spondylitis metrology index
Cox-2: Cyclooxygenase-2
CRP: C-reactive protein
DFI: Dougados functional index
DMARDs: Disease modifying anti-rheumatic drugs
EQ-5D: EuroQoL 5-dimension
ESR: Erythrocyte sedimentation rate
GRC: Global rating of change
HADS: Hospital anxiety and depression scale
HRQoL: Health-related quality of life
IBD: Inflammatory bowel disease
LDQ: Leeds disability questionnaire
NSAIDs: Non-steroidal anti-inflammatory drugs
PsA: Psoriatic arthritis
QALYs: Quality-adjusted life-years
QoL: Quality of life
SD: Standard deviation
SES: Standardized effect size
SF-36: 36-Item short-form
SpA: Spondyloarthritis
SRM: Standardized response mean
VAS: Visual analogue scale
WD: Work disability
